# Supplementary material for: A general and efficient representation of ancestral recombination graphs
Source: bioRxiv. 2024 Apr 23:2023.11.03.565466. Originally published 2023 Nov 4. Preprint. [Version 2] doi: 10.1101/2023.11.03.565466 (PMC10635123; doi:10.1101/2023.11.03.565466)
Supplement: 1 [file NIHPP2023.11.03.565466V2-supplement-1.pdf]

bioRxiv preprint doi: https://doi.org/10.1101/2023.11.03.565466; this version posted April 23, 2024. The copyright holder for this preprint (which was not certified by peer review) is the author/funder, who has granted bioRxiv a license to display the preprint in perpetuity. It is made available under aCC-BY 4.0 International license.

Supplementary Material

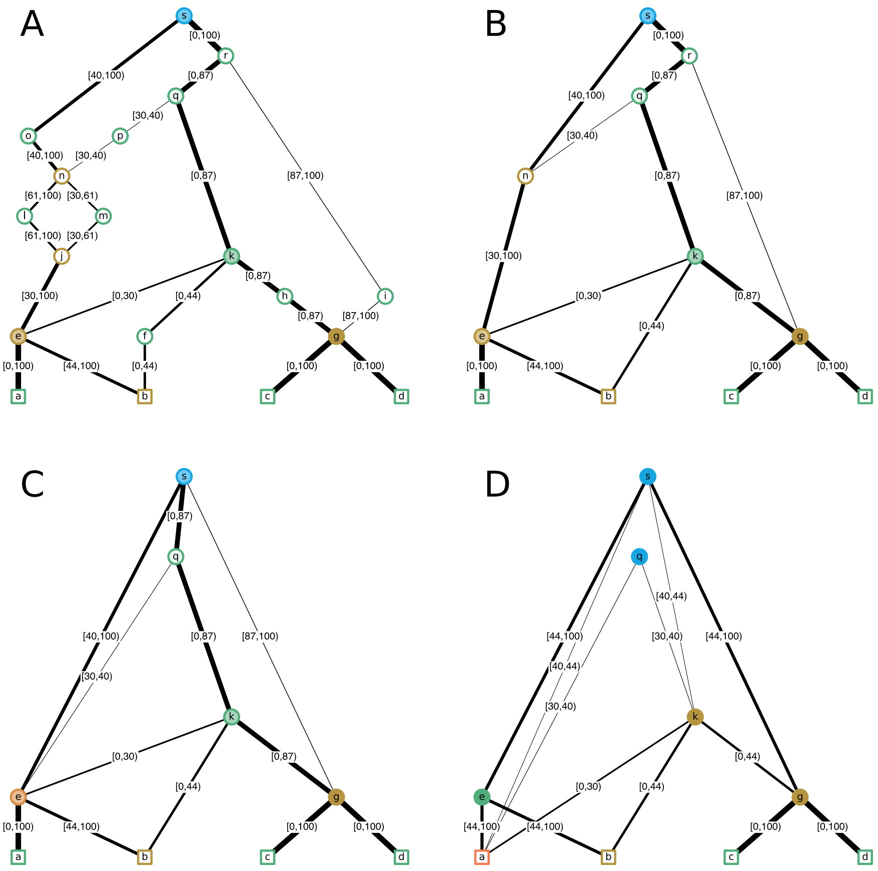

Figure S1: Example ARGs Fig. 5A–D, with edges annotated with inheritance intervals.
