# Supplementary material for: A general and efficient representation of ancestral recombination graphs
Source: bioRxiv. 2023 Nov 4:2023.11.03.565466. Preprint. [Version 1] doi: 10.1101/2023.11.03.565466 (PMC10635123; doi:10.1101/2023.11.03.565466)
Supplement: Supplementary file 1 [file NIHPP2023.11.03.565466v1-supplement-1.pdf]

bioRxiv preprint doi: https://doi.org/10.1101/2023.11.03.565466; this version posted November 4, 2023. The copyright holder for this preprint (which was not certified by peer review) is the author/funder, who has granted bioRxiv a license to display the preprint in perpetuity. It is made available under aCC-BY 4.0 International license.

Supplementary Material

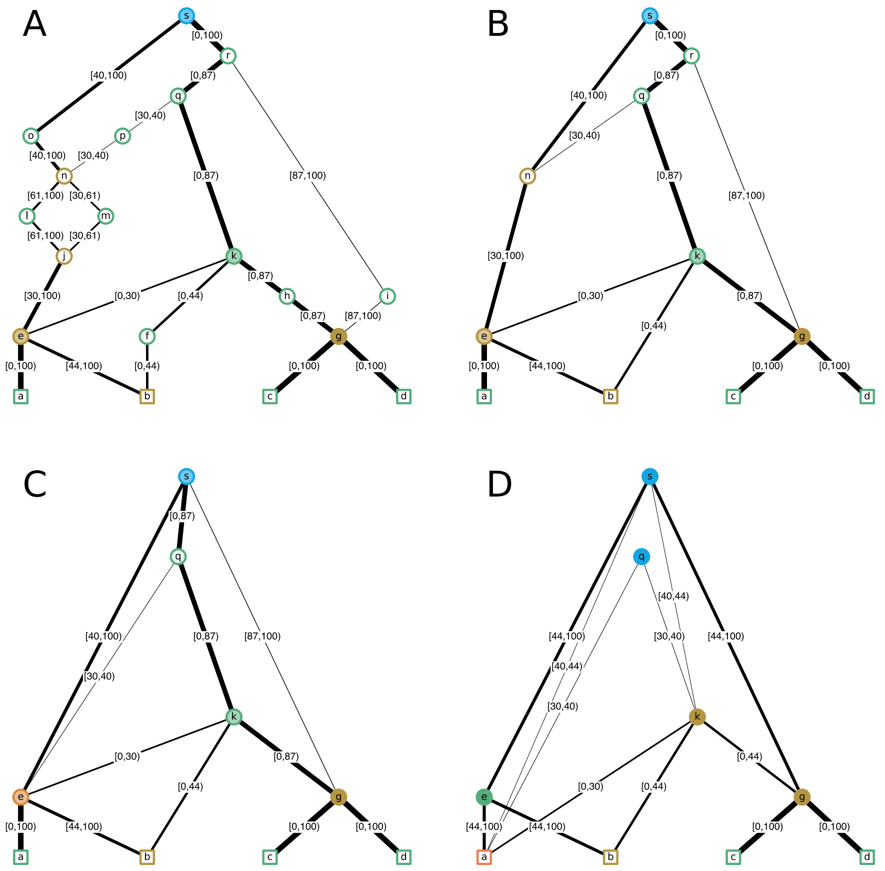

Figure S1: Example ARGs Fig. 4A–D, with edges annotated with inheritance intervals.
